# Multipurpose Passive Surveillance of Bat-Borne Viruses in Hungary: Lyssaviruses and Filoviruses in Focus

**DOI:** 10.3390/ani15243590

**Published:** 2025-12-14

**Authors:** Anna Szabó, Zsófia Lanszki, Gábor Kemenesi, Alexandra Nándori, Péter Malik, Krisztián Bányai, Henrik Fülöp Károlyi, Ágnes Nagy, Endre Sós, Pavle Banović, Tamás Görföl

**Affiliations:** 1 National Laboratory of Virology, Szentágothai Research Centre, University of Pécs, 7624 Pécs, Hungary; szabo.anna@pte.hu (A.S.); kemenesi.gabor@pte.hu (G.K.); karolyi.henrik@pte.hu (H.F.K.); tamas@gorfol.eu (T.G.); 2 Institute of Biology, Faculty of Sciences, University of Pécs, 7624 Pécs, Hungary; 3 Department of Virology, Directorate of the Veterinary Diagnostic Laboratory, National Food Chain Safety Office, 1143 Budapest, Hungary; nandoria@nebih.gov.hu (A.N.); malikp@nebih.gov.hu (P.M.); 4 Department of Medical Biology, Medical School, University of Pécs, 7624 Pécs, Hungary; bkrota@hotmail.com; 5 Department of Pharmacology and Toxicology, University of Veterinary Medicine, 1078 Budapest, Hungary; 6 Budapest Zoo and Botanical Garden, 1146 Budapest, Hungary; agnesnagyemail@gmail.com (Á.N.); drsos.endre@zoobudapest.com (E.S.); 7 Department of Microbiology with Parasitology and Immunology, Faculty of Medicine in Novi Sad, University of Novi Sad, 21000 Novi Sad, Serbia; pavle.banovic@mf.uns.ac.rs; 8 Diagnostics and Laboratory Research Task Force, Balkan Association for Vector-Borne Diseases, 21000 Novi Sad, Serbia; 9 Department for Microbiological and Other Diagnostics, Pasteur Institute Novi Sad, 21000 Novi Sad, Serbia

**Keywords:** surveillance, bat, lyssavirus, disease, One Health, filovirus, Europe

## Abstract

Bats are vital parts of ecosystems but also act as important reservoirs of viruses that can infect humans. Studying bat-borne viruses is essential for preventing spillover events, and passive surveillance offers a promising, bat-friendly approach. Among these viruses, lyssaviruses are a major public health concern because infection in humans is almost always fatal. In Europe, the European bat lyssavirus type 1 (EBLV-1) is the most widespread. We aimed to expand the Hungarian rabies surveillance system into a One Health-based passive surveillance framework for multiple bat viruses, using lyssaviruses and filoviruses as examples. Dead-found or injured bats that died despite care were collected for examination. In total, 208 bats from 15 species were tested for lyssaviruses and Lloviu virus. Three serotine bats (*Cnephaeus serotinus*, formerly *Eptesicus*) tested positive for EBLV-1, and complete viral genomes were obtained. All sequences belonged to the EBLV-1a lineage, closely related to previously described Hungarian strains. All bats tested negative for Lloviu virus. These results confirm the feasibility of passive surveillance for detecting bat-borne viruses and highlight the importance of collaborative monitoring systems for future zoonotic outbreak preparedness.

## Simple Summary

This study describes the establishment of a new passive surveillance system to monitor diseases in bats in Hungary, and reports the first scientific results obtained using this approach. We retrospectively screened bat specimens collected over six years to assess the presence of viruses with zoonotic potential in Europe. European bat lyssavirus type 1 was detected in three serotine bats, while all samples tested negative for Lloviu virus, the only filovirus known to circulate enzootically in Europe. These findings provide new insights into ongoing viral surveillance efforts and highlight the importance of systematic retrospective screening in European bat populations.

## 1. Introduction

Global anthropogenic changes resulting in biodiversity loss and ecosystem degradation contribute to the emergence and spread of diseases [1]. One Health is an integrated approach that seeks to balance human, animal and ecosystem health by combining traditional disease-based surveillance with monitoring the factors driving disease emergence, thereby enhancing the prevention and mitigation of spill-over events [2]. It is known that preventing these spill-over events is much more cost-effective than relying on response activities [3].

Bats are mammals with unique adaptations and attributes that play critical roles in ecosystems, making them a key focus of One Health research. These animals possess a highly specialized immune system that enables them to harbor diverse pathogens, including viruses, while typically remaining asymptomatic [4,5]. Several viral groups contain large numbers of bat-borne viruses, like the *Paramyxoviridae* and *Coronaviridae* families, while other groups exhibit much lower genetic diversity. A good example of the latter is the *Filoviridae* family, that contains few but very important members, like Ebola- and Marburgviruses. The above mentioned viruses caused many deadly human outbreaks worldwide, and this has led to a growing interest in the role of bats as natural reservoirs for zoonotic diseases, making them a vital link in understanding the intricate connections between animal, human, and environmental health within the One Health framework [6,7,8].

Among the most well-known viruses are lyssaviruses, a diverse group of neurotropic viruses that can cause rabies, an invariably fatal acute encephalitis in mammals. These negative single-stranded, non-segmented RNA viruses are members of the *Rhabdoviridae* family in the order *Mononegavirales* [9]. There are 18 described species in the genus *Lyssavirus*, according to the International Committee on Taxonomy of Viruses (ICTV). Lyssaviruses can be divided into three phylogroups. Phylogroup I contains *Lyssavirus australis* (ABLV), *L. aravan* (ARAV), *L. bokeloh* (BBLV), *L. duvenhage* (DUVV), *L. hamburg* (EBLV-1), *L. helsinki* (EBLV-2), *L. gannoruwa* (GBLV), *L. irkut* (IRKV), *L. kotalahti* (KBLV), *L. khujand* (KHUV), *L. formosa* (TWBLV) [10], and *L. rabies* (RABV) viruses, while *L. lagos* (LBV), *L. mokola* (MOKV), and *L. shimoni* (SHIBV) are members of phylogroup II [11]. Based on phylogenetic distance, the most genetically distinct lyssaviruses are *L. ikoma* (IKOV), *L. lleida* (LLEBV), and *L. caucasicus* (WCBV), forming the putative phylogroup III. Lyssaviruses can enter the body through multiple routes, with the most common being through a bite from an infected animal [12,13,14]. Once the clinical signs are present, there is an almost 100% fatality rate. Therefore, pre- and post-exposure vaccines and immune globulin treatments are the only options for disease prevention. Rabies vaccines have been shown to provide protection against lyssaviruses within Phylogroup I, and some vaccines may offer partial cross-protection against viruses belonging to Phylogroups II and III [15,16]. In most European countries, it is advised that most individuals who are professionally or recreationally exposed to bats undergo pre-exposure immunization against rabies [17].

Since EBLV was first isolated in Germany in 1954 [18] and Yugoslavia in 1955 [19], more than a thousand rabid bat cases have been reported, especially in the western part of Europe (e.g., [20,21,22]). EBLV-1 has been linked to *Cnephaeus serotinus* and *C. isabellinus*, EBLV-2 to *Myotis daubentonii* and *M. dasycneme*, while LLEBV and WCBV to *Miniopterus schreibersii* [23]. Most lyssavirus-infected bats have been discovered in Northern and Central Europe, specifically in the UK, Germany, Finland, and Denmark [20,24,25,26]. This is probably due to the more extensive surveillance in these areas rather than significant differences in epidemiology [27], and thus, differences in sampling intensity should be considered when interpreting current data.

Lloviu virus (LLOV), the only known European member of the *Filoviridae* family, was first identified during large bat die-off events on the Iberian Peninsula in the early 2000s [28]. The virus re-emerged in Hungary in the 2010s and has since been detected in several European countries, including Italy [29,30,31]. Its host range, geographic distribution, ecological and epidemiological characteristics, and zoonotic potential remain unknown, making further research on this Ebola-related virus critically important.

Combined serological and virus detection methods have revealed that at least three lyssaviruses are in circulation in bat populations in Hungary; however, with molecular methods, only EBLV-1 has been detected from a single species, *C. serotinus* [32]. A wide-range serological study of *Miniopterus schreibersii* revealed that bats from Hungary were infected with WCBV and LLEBV [22]. There are 29 bat species in Hungary, and several of them are hosts for diverse viruses, e.g., filoviruses, adenoviruses, picornaviruses, caliciviruses, astroviruses, herpesviruses, and coronaviruses as revealed with targeted molecular screenings [32,33,34,35].

In the context of wildlife, passive surveillance involves virological testing of animals that have died as a result of injury or debilitation [36]. This method is of paramount importance, as some of the animals found may have been infected with viruses that are responsible for the disease signs. Clinically asymptomatic rabies infections have also been observed in bats, highlighting the importance of thorough surveillance [37,38]. Passive surveillance systems for bat viruses in Europe focus on detecting diseases, such as European bat lyssaviruses in naturally deceased or ill bats, crucial for understanding zoonotic risks. Countries like Germany and France have well-established passive surveillance programs, monitoring rabies-related viruses in bats since the late 20th century. For instance, Germany’s surveillance has detected both EBLV-1 and EBLV-2, contributing significantly to phylogenetic studies of these pathogens [39]. Researchers in France noted an unusual bat mortality event and several EBLV cases in the frame of their passive surveillance system [40]. The UK employs a system that has tested over several thousand bats for EBLV since the 1980s, demonstrating the importance of ongoing surveillance to track viral distribution [41] and resulting in the identification of the first case of EBLV-1 in the country [26]. Importantly, findings emphasize the interconnectedness of wildlife health and human risk, supporting One Health principles. By identifying patterns of viral circulation, these systems enable early warnings for public health interventions, as recently observed in the Netherlands [42]. 

In Hungary, rabies-suspected bats were subjected to investigation at the National Reference Laboratory for Rabies of the National Food Chain Safety Office, Budapest. This system provided a safe protocol to prevent human cases but did not allow a wide-range passive surveillance of bats and to search for other pathogens. Here we present our passive surveillance system of Hungarian bats and the first results regarding viral disease monitoring.

## 2. Materials and Methods

### 2.1. Samples 

We examined bat carcasses from Hungary, Central Europe that had died due to various factors such as disease, human activities, and other threats. The specimens primarily came from the rescue centre of the Budapest Zoo and Botanical Garden, other rescue centres, national parks, or veterinary clinics, collected by various experts, bat researchers and conservationists between 2018 and 2024. Altogether 15 species comprising 208 individuals were included in the study, serotine bat (*Cnephaeus serotinus*) (n = 26), Savi’s pipistrelle (*Hypsugo savii*) (n = 12), Daubenton’s bat (*Myotis daubentonii*) (n = 4), greater mouse-eared bat (*M. myotis*) (n = 4), whiskered bat (*M. mystacinus*) (n = 1), Natterer’s bat (*M. nattereri*) (n = 1), lesser noctule (*Nyctalus leisleri*) (n = 1), common noctule (*N. noctula*) (n = 43), Kuhl’s pipistrelle (*Pipistrellus kuhlii*) (n = 94), common pipistrelle (*P. pipistrellus*) (n = 3), Nathusius’ pipistrelle (*P. nathusii*) (n = 3), grey long-eared bat (*Plecotus austriacus*) (n = 7), brown long-eared bat (*P. auritus*) (n = 2), lesser horseshoe bat (*Rhinolophus hipposideros*) (n = 1), parti-coloured bat (*Vespertilio murinus*) (n = 6) (Table 1). Until further processing, the bat carcasses were stored at −80 °C. As all bat species in Hungary are protected, the relevant authorities issued research and sample collection permits to the Hungarian Natural History Museum (PE-KTF/736-6/2017, PE-KTFO/329-16/2019, PE-KTFO/1568-18/2020, PE-KTFO/1403-3/2022). Bat species names were used according to the latest taxonomic reference and *serotinus* was placed in *Cnephaeus* instead of its former genus, *Eptesicus* [43].

### 2.2. Bat Necropsy

To ensure maximum safety, the necropsy of the bats was carried out under BSL-2+ conditions. Accordingly, the personal protective equipment (PPE) included double gloves (with the inner layer sealed), laboratory scrubs and a laboratory coat worn on top.

The dissected organs were as follows: brain, lungs, heart, liver, spleen, kidneys, rectum, testicles and muscle. Dissections were performed in a consistent order, beginning with the brain and subsequently proceeding to the other organs, with sterile scissors and tweezers used throughout. To further reduce the likelihood of cross-contamination, instruments were exchanged after the brain dissection, before proceeding with the remaining organs. Each sample was divided and placed into two 1.5 mL Eppendorf tubes without any preservatives or snap freezing and stored directly at −80 °C until further processing. For the testicle samples, one testis was frozen and stored natively at −80 °C, while the other testis was fixed in 6% formaldehyde for subsequent research purposes. External and internal characteristics (e.g., sex, age, condition of organs), abnormalities visible on the animals (e.g., injuries, weak condition) were recorded before and during the necropsy.

### 2.3. Nucleic Acid Extraction and PCR Reactions

Phosphate-buffered saline (PBS, 300 μL) was added to approximately half of the brain and lung samples and were homogenized for three minutes using a TissueLyser LT (Qiagen, Hilden, Germany). The total RNA was extracted using Monarch Total RNA Miniprep Kit (New England Biolabs, Ipswich, MA, USA) and finally eluted in 40 µL of nuclease-free water.

Following nucleic acid extraction, the brain samples were screened with a universal RT-nPCR targeting a conserved region of the N (nucleoprotein) gene for the representatives of the genus *Lyssavirus*. First round of the PCR was used with the Luna Universal One-Step RT-qPCR Kit (New England Biolabs, Ipswich, MA, USA) with the reaction setup as follows: at 55 °C for 10 min, and 95 °C for 1 min, followed by 30 cycles of 95 °C for 10s, 53 °C for 30s, 60 °C for 1 min, finally 60 °C for 6 min, and 4 °C ∞. The following primer sets were used: GRAB1F and GRAB1R [44]. The second round was used with GoTaq G2 Flexi DNA Polymerase Kit (Promega, Madison, WI, USA) with endpoint PCR. The reaction setup for the second round: 94 °C for 2 min, followed by 30 cycles of 94 °C for 1 min, 53 °C for 1 min, and 72 °C for 1 min, finally 72 °C for 5 min, and 4 °C ∞. The following primers were used: GRAB2F and GRAB2R [44]. The PCR products were analysed by standard gel electrophoresis using a 1.5% agarose gel. In case of lyssavirus positivity, nucleic acid purification and PCR reaction were conducted on all the necropsied organs (heart, lungs, liver, kidneys, spleen, testicle), using the same method.

The lung samples were screened with a Lloviu virus-specific real-time RT-PCR system. The PCR was used with the qRT-PCR Brilliant III Probe Master Mix (Agilent Technologies, Santa Clara, CA, USA) with the reaction setup as follows: at 50 °C for 10 min, and 95 °C for 3 min, followed by 50 cycles of 95 °C for 5 s, 60 °C for 30 s [30].

### 2.4. Viral Enrichment and RNA Extraction

A viral enrichment process was used to increase the proportion of viral reads. We followed a modified version of the method published by Conceição-Neto et al. [45]. The PCR-positive homogenates were centrifuged at 16,000× *g* for 10 min. From the sample, 160 μL supernatant was filtered through a 0.45 μm Ultrafree-CL Centrifugal Filter (Merck Millipore, Burlington, MA, USA). 150 μL of the sample was treated for 2 h at 37 °C with a cocktail of 1 μL micrococcal nuclease (New England Biolabs, Ipswich, MA, USA), 2 μL of benzonase (Merck Millipore, Burlington, MA, USA), 4.5 µL Turbo DNase and 15.5 µL Turbo DNase Buffer (Invitrogen, Waltham, MA, USA), and extracted with the Direct-Zol RNA MiniPrep Kit (Zymo Research, Irvine, CA, USA).

### 2.5. Illumina Sequencing

RNA library was generated using the NEBNext Ultra II Directional RNA Library Prep for Illumina (New England Biolabs, Ipswich, MA, USA). Briefly, 10 ng of total RNA was used as input for the fragmentation step, and the cDNA generation was performed using random primers. Thereafter, the cDNA was end-prepped and adapter-ligated, then the library was amplified according to the manufacturer’s instructions. The quality of the libraries was checked on an Agilent 4200 TapeStation System using D1000 Screen Tape (Agilent Technologies, Santa Clara, CA, USA), and the quantity was measured on Qubit 3.0 (Thermo Fisher Scientific, Waltham, MA, USA). Illumina sequencing was performed on a NovaSeq 6000 instrument (Illumina, San Diego, CA, USA) with 2 × 151 run configuration.

### 2.6. Phylogenetic Analysis 

The Illumina sequencing reads were aligned to the closest complete rabies virus genomes in Geneious Prime v2024.0.4. After manual corrections, the complete genomes were obtained and deposited in the NCBI GenBank under PV454706-PV454708 accession numbers. They were aligned with other complete Lyssavirus genomes downloaded from the GenBank using MAFFT v7.505 software. IQ-TREE v2.3.6 [46] was used to find the best fitting substitution model (GTR + F + G4 and JC in case of EBLV-1 and Lyssavirus trees, respectively) and generate a Maximum Likelihood tree with 1000 bootstrap support. The tree was visualized using iTol v7.0 online [47].

## 3. Results

### 3.1. A Passive Surveillance System to Detect Bat-Borne Viruses

**Figure 1:**
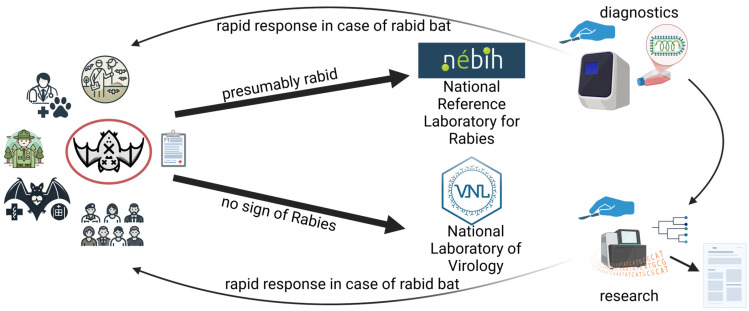
Schematic figure of the implemented passive surveillance system of bat-borne diseases.

To date, no passive surveillance has been conducted in Hungary for the virological examination of deceased bats. Therefore, the newly established passive surveillance system in Hungary operates through two main laboratories: the Department of Virology at the Directorate of the Veterinary Diagnostic Laboratory of the National Food Chain Safety Office (=National Reference Laboratory for Rabies, located in Budapest, the capital) and the National Laboratory of Virology at the University of Pécs (situated in Pécs, southern Hungary) (Figure 1). 

Animal rescue centres, which receive a variety of animals including bats, are primary contributors of specimens. Another significant source of bat submissions come from bat researchers, who are often the first professionals to interact with these animals. In collaboration with bat rehabilitation centres, we organized workshops, prepared documentation, and provided training to educate researchers about the potential health hazards associated with bats. Veterinarians and national park staff, including rangers, were also integrated into the surveillance network due to their frequent encounters with injured or deceased bats.

It is essential to collect as much information as possible about the bats and the circumstances of their discovery. This information serves two critical purposes: it allows for timely contact with relevant contact persons in case a bat tests positive for rabies, it aids decision about where to send the bats for investigation, and it can also support further research efforts.

If a bat is suspected of being infected with the rabies virus, it is sent to the National Reference Laboratory for Rabies. Investigations at the National Reference Laboratory for Rabies are conducted promptly, particularly in cases involving potential human exposure. If rabies infection is not suspected, the bat is sent to the National Laboratory of Virology at the University of Pécs. Here, additional viral groups are investigated besides lyssaviruses.

### 3.2. Lyssavirus Survey

During passive surveillance of lyssaviruses in bats collected and tested in Hungary between 2018 and 2024, three out of 208 brain samples were positive for lyssavirus RNA. All positive samples were detected in serotine bats. We also tested the lungs, hearts, livers, spleens, and kidneys of two positive bats, as well as a testicle from a male bat, for the presence of lyssavirus. All the tested organs of the female bat were positive, except the spleen tissue. In the case of the male bat, all the tested organs, including the testicle, were positive for lyssavirus.

The (near) full-length genomic sequence of all three viral RNA positive samples could be determined from the Illumina sequencing outputs. Remapping of reads to the newly generated genomes resulted in mean coverages 23452.2X (1,650,328 reads), 76.1X (5810 reads), and 91.4X (7296 reads) in case of PC454706, PV454707, and PV454708, respectively. The finalized consensus genomes were as follows: PV454706, 11966 nt; PV454707, 11966 nt; PV454708, 11931 nt. The genomic structure of the three Hungarian sequences was similar to those of other EBLV-1a strains (N, 1356 nt, 452 aa; P, 897 nt, 299 aa; M, 609 nt, 203 aa; G, 1575 nt, 525 aa; L, 6384 nt, 2128 aa). The PV454708 sequence was incomplete, with a 23 nt gap in the N gene. The order and length of genes were conserved and virtually identical to the reference EBLV-1 strain, RV9, an German isolate from 1968 (GenBank #, NC_009527). No other viruses were found in the samples of these three bats.

**Figure 2:**
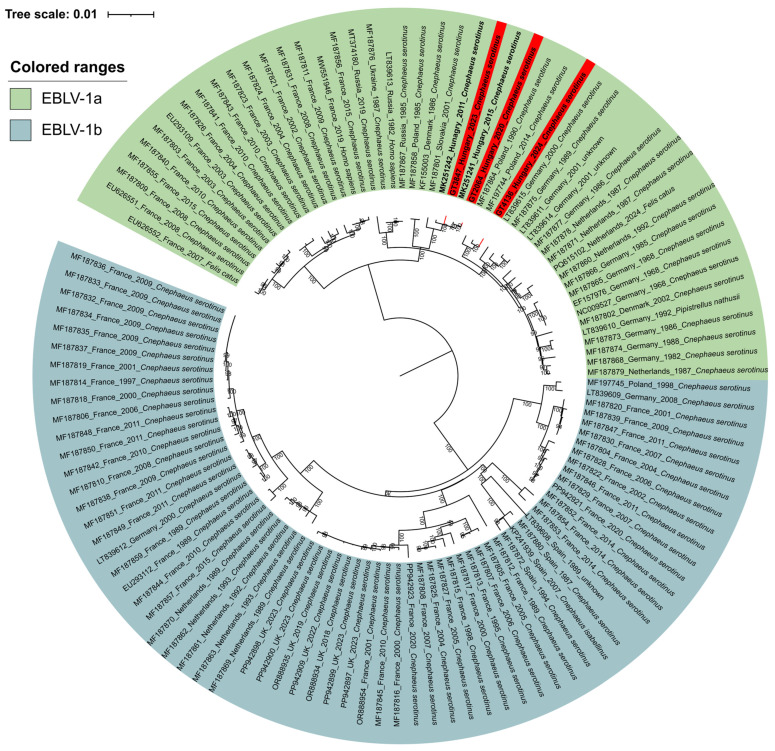
Maximum-Likelihood phylogenetic tree was constructed based on 116 complete European bat lyssavirus-1 (EBLV-1) genomes. Hungarian sequences identified in this study are highlighted with a red background and bold font, while earlier Hungarian sequences with bold font.

The phylogenetic analysis of the complete genomes confirms the results obtained from the partial sequence fragments, indicating that the three viruses belong to the EBLV-1a phylogenetic cluster (Figure 2). The genomes showed the highest similarity to EBLV-1a viruses previously isolated from serotine bats in Hungary in 2011 and 2015 [32] and clustered with sequences of EBLV-1a retrieved from *C. serotinus* from Slovakia and Poland between 1990 and 2014. Two strains (GT2064 from 2020 and GT3847 from 2023) clustered with other Hungarian strains isolated in 2011 and 2015, while the third strain (GT4136 from 2024) clustered with EBLB-1a strains detected in 1990 and 2014 in Poland. The genome wide sequence homology values between the two sister branches were ~98.5%, or so. This pattern of tree topology suggests that EBLV-1a strains identified thus far in Hungary may have originated from diverse geographic location through distinct flyways of migrating bats.

**Figure 3:**
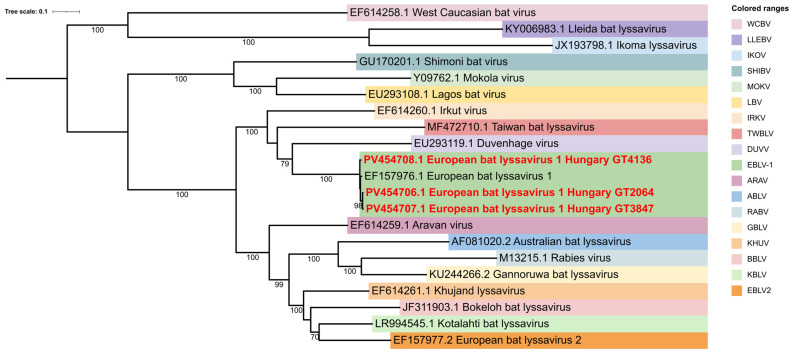
Maximum-Likelihood phylogenetic tree was constructed from 21 complete genomes representing 18 Lyssavirus species. Hungarian sequences identified in this study are highlighted in red.

We also created a phylogenetic tree that provides a clearer overview of the diversity across all lyssavirus species, highlighting the evolutionary relationship that define each major lineage (Figure 3). Our three sequences consistently clustered with EBLV-1, forming a well-supported subgroup that reflects their close genetic affinity to this lineage. Notably, their position also placed them near several geographically distinct viruses; DUVV, TWBLV and IRKV, suggesting shared ancestral origins while maintaining characteristic separation.

Although we did not receive exact collection localities for all bats, the habitat preference of all species is well documented, and most bat species from this study are linked to urban habitats. Thus, over 90% of those are representatives of partly (*Nyctalus noctula*, *Myotis myotis*, *Rhinolophus hipposideros*, *Myotis daubentonii*, *Pipistrellus nathusii*, *Pipistrellus pipistrellus*) or exclusively building-dweller bat species (*Cnephaeus serotinus*, *Pipistrellus kuhlii, Hypsugo savii*, *Vespertilio murinus*, *Plecotus austriacus*) where human-bat encounters are more probable.

### 3.3. Surveillance Data on EBLV-1a Positive Bats

On 4 June 2020, near Sukoró, Hungary, a female bat was pulled underwater by a frog. Serendipitously, pedestrians witnessed the incident and managed to rescue the bat. The animal was subsequently transferred to the Animal Rescue Centre of the Budapest Zoo and Botanical Garden for veterinary care. No clinical signs of rabies were observed for several months. The bat exhibited normal behaviour during autumn and winter. Hibernation was conducted under controlled conditions, with monthly monitoring and feeding. On 1 March 2021, its weight was recorded at 26.2 g, yet by 3 April 2021, it had significantly declined to 16 g. Despite continued veterinary care, the bat died on 12 April 2021, over ten months after its initial admission to the rehabilitation centre. The second case originated from 29 September 2023, in Budapest. The male serotine bat was discovered trapped inside a police station. The animal was promptly taken to a veterinary clinic for assessment and care. However, despite medical attention, the bat was found deceased the following day. The third serotine was found on 2 July 2024, in Budapest. The bat was weak and there was a haemorrhage on the right forearm. It was fed for four days, but on 6 July, it refused both food and water. It was euthanized on 8 July. No human contacts were reported, however, post-exposure rabies prophylaxis was recommended in the latter two cases, as the bats died shortly after being brought them into expert care.

### 3.4. Lloviu Virus Survey

In addition, for the lyssavirus analyses, lung tissue samples from all bats were included in the surveillance and were tested for the presence of Lloviu virus. All samples were negative, indicating no evidence of Lloviu virus in the examined bat species during the study. These findings are consistent with current knowledge that Lloviu virus has so far only been detected in Schreibers’s bats (*Miniopterus schreibersii*) and has not been reported from other bat species.

## 4. Discussion

Lyssaviruses have caused rabies in humans for centuries, leading researchers into creating effective strategies to prevent infection. Passive surveillance has a major importance in studying viruses circulating in the bat fauna, and as most bats found are from urban areas, this especially applies for the study of bats close to humans. This method has been used successfully by several countries in Europe since the 1980s [20,41,48,49]. However, these surveillance programs often concentrate only on lyssaviruses and other viral groups are studied occasionally. Although no other viruses were detected in this study, we conducted a proof-of-concept investigation to identify potential new hosts of Lloviu virus. Lloviu virus has been associated with mortality events in Schreiber’s bats, although not yet conclusively [28]. We believe that reporting negative findings is also important, as it contributes to a more complete understanding of viral ecology. Thus, the assumption that it may occur in animals collected during passive surveillance and provide useful samples for understanding the host spectrum outside of Schreiber’s bats is a novel approach to Lloviu virus research.

Our main goal was to develop a new surveillance system based on the well-performing Hungarian rabies surveillance program, to be able to respond rapidly to rabies cases as well as to be able to study the bat viruses circulating in Hungary more deeply. Based on the One Health concept, which acknowledges that human health is strongly linked to the health of animals, plants and all its environment, the integrated work of different sectors and regions should rely on detection and response infrastructures [50]. During our research, we were able to work together in a collaborative way with many bat experts, rescue centres, national park directorates and veterinary clinics. This acquaintance and cooperation between our research facilities and these partners gives the system an unparalleled opportunity to effectively study the bat-borne viruses—including lyssaviruses and Lloviu virus—circulating in Hungarian bats. The passive surveillance system that we aimed to establish in Hungary is particularly valuable and important because it fills a critical gap that has not yet been addressed, providing a solution to a long-standing deficiency.

Based on our experience, recording detailed information about the circumstances in which a bat is found can be crucial, as it may indicate the presence of pathogens. This should also include information on the contact person, to be able to reach them in case of positivity. In our study, we were able to include 15 of the 29 bat species found in Hungary. Previous studies have shown that successful passive surveillance is dependent on recovering bats from a geographical range that reflects their natural distribution [9].

Raising awareness of effective rabies control measures, both among public health professionals and the public, to reduce the risk of human infection is a very important task of this system. Misconception or misinformation are a significant issue in the realm of the public’s understanding of how bats pose a risk to humans, underscoring the necessity of enhanced risk communication strategies and avoiding negative framing of bats [51], while remaining vigilant in cases of bite exposure [42,52]. We also wanted to make suggestions to bat conservationists, chiropterologists, and handlers on the honest and accurate risks of zoonotic virus spillover from bats and how to prevent such cases. Cautious handling is one of the most crucial parts of dealing with these animals, where personal protective equipment, such as gloves, masks, and in special cases hazard suits can prevent unfortunate accidents. Furthermore, individuals at rescue centres and bat researchers should be vaccinated against rabies and this is also recommended for any other person who is working with bats regularly (e.g., veterinarians, biologists, and other professionals), and we additionally recommend periodic antibody titre testing to verify adequate post-vaccination immunity.

Although several viral groups are investigated within our passive surveillance system, our case study focused on the results of our lyssavirus and Lloviu virus survey. The lyssavirus findings are consistent with previous studies, as the serotine bat is the main host for EBLV-1 [20]. The virus is still present and circulating amongst these animals in Hungary, therefore it is crucial to continue the monitoring to gain a better understanding of the virus’s prevalence. In the frame of our study, we have found three serotine bats that were infected with EBLV-1. The complete viral genomes were retrieved from the brains of these animals, all belonging to the EBLV-1a strain. This is in line with the other two Hungarian strains from rabid bats found earlier [32]. The new viruses were found to be closely related to those previously detected in Hungary, Poland, and Slovakia. These closely related lineages were also derived from *Cnephaeus serotinus* bats, comprising Polish isolates collected in 1990 and 2014, as well as a Slovak strain recovered in 2001. Long-term surveillance efforts targeting lyssaviruses in European bat populations have been ongoing since 1977, consistently revealing that *Cnephaeus serotinus* is the primary reservoir species. Throughout these surveillance programs, most detected infections across Europe have been attributed to European bat lyssavirus type 1 (EBLV-1), underscoring its widespread circulation and long-standing presence within serotine bat populations [24,37,53,54,55]. Examination of the other organs of two positive bats revealed discrepancy in lyssavirus presence between the tested organs (spleen was positive in one animal and negative in the other). This may be the result of different tissue tropism within the host or other factors [56].

Our findings align with recent European surveillance data on lyssaviruses, highlighting their sporadic but widespread circulation across the region. In contrast, only limited surveillance data are currently available for filoviruses in European bats [30,57] underscoring the need for further surveillance. Tracking and monitoring human-bat interactions are essential components of the One Health approach, helping to predict and prevent potential outbreaks or pandemics particularly in Europe, where these interactions are frequent [52,55,58,59]. To achieve this goal, we aimed to establish a passive surveillance system for bat-borne viruses in Hungary, and to study the bat lyssavirus situation in the country with the help of the public and through the collaboration of healthcare and research institutions. Moreover, considering the results of our study, we hope this paper will serve as a recommendation for both the public and professionals working with bats (e.g., veterinarians, conservation biologists), emphasizing that caution is one of the most important aspects of handling these animals. A limitation of the study is that not all bat species present in Hungary could be included in the sampling, and the number of collected samples for each species fluctuated between years, which may influence the representativeness and comparability of the results.

## 5. Conclusions

Collectively, this study represents the implementation of a One Health approach surveillance system in a Central European country. The future objective is to expand the research on a broader scale, investigate the presence of additional viruses, or investigate emerging bat-related infectious diseases within the framework of the established passive surveillance system.

## Figures and Tables

**Table 1:** Annual number of bat species and carcasses examined between 2018–2024 in Hungary. Typical habitats for each species are also indicated.

Species	*Cser*	*Hsav*	*Mdau*	*Mmyo*	*Mmys*	*Mnat*	*Nlei*	*Nnoc*	*Pkuh*	*Pnat*	*Ppip*	*Paur*	*Paus*	*Rhip*	*Vmur*	∑
Year/Habitat	U	U	U/R	U/R	R	R	R	U (R)	U	R (U)	R (U)	R	U	U	U	
2018	-	-	-	-	-	-	1	-	1	-	-	-	-	-	-	2
2019	-	-	-	-	1	-	-	1	1	-	-	-	-	-	-	3
2020	1/1	3	1	3	-	-	-	3	2	-	-	-	-	-	-	13
2021	2	-	-	-	-	1	-	21	12	-	1	-	2	-	4	43
2022	6	5	1	-	-	-	-	7	59	1	-	-	3	1	1	84
2023	7/1	-	-	1	-	-	-	6	12	2	1	1	1	-	-	31
2024	1/1	1	1	-	-	-	-	-	-	-	-	-	-	-	-	3
2018–2024 (no specific date)	9	3	1	-	-	-	-	5	7	-	1	1	1	-	1	29
∑	26/3	12	4	4	1	1	1	43	94	3	3	2	7	1	6	208

Abbreviations. *Cser*—*Cnephaeus serotinus*, *Hsav*—*Hypsugo savii*, *Mdau*—*Myotis daubentonii*, *Mmyo*—*Myotis myotis*, *Mmys*—*Myotis mystacinus*, *Mnat*—*Myotis nattereri*, *Nlei*—*Nyctalus leisleri*, *Nnoc*—*Nyctalus noctula*, *Pkuh*—*Pipistrellus kuhlii*, *Pnat*—*Pipistrellus nathusii*, *Ppip*—*Pipistrellus pipistrellus*, *Paur*—*Plecotus auritus*, *Paus*—*Plecotus austriacus*, *Rhip*—*Rhinolophus hipposideros*, and *Vmur*—*Vespertilio murinus*. U, urban; R, rural.

## Data Availability

All the sequences generated have been deposited in NCBI GenBank database with accession numbers PV454706, PV454707, and PV454708. The datasets generated during and/or analysed during the current study are available from the corresponding author upon request.

## Abbreviations

The following abbreviations are used in this manuscript:

ABLV	*Lyssavirus australis*
ARAV	*Lyssavirus aravan*
BBLV	*Lyssavirus bokeloh*
cDNA	Complementary Deoxyribonucleic Acid
DUVV	*Lyssavirus duvenhage*
EBLV	*European bat lyssavirus*
EBLV-1	*Lyssavirus hamburg*
EBLV-1a	*European bat lyssavirus 1a strain*
EBLV-2	*Lyssavirus helsinki*
GBLV	*Lyssavirus gannoruwa*
ICTV	International Committee on Taxonomy of Viruses
IKOV	*Lyssavirus ikoma*
IRKV	*Lyssavirus irkut*
KBLV	*Lyssavirus kotalahti*
KHUV	*Lyssavirus khujand*
LBV	*Lyssavirus lagos*
LLEBV	*Lyssavirus lleida*
LLOV	*Cuevavirus lloviuense*
MOKV	*Lyssavirus mokola*
N gene	Nucleoprotein gene
NCBI	National Center for Biotechnology Information
PBS	Phosphate-buffered Saline
Pan-Lyssa RT-nPCR	Pan-Lyssavirus reverse transcription nested polimerase chain reaction
RABV	*Lyssavirus rabies*
RNA	Ribonucleic Acid
SHIBV	*Lyssavirus shimoni*
TWBLV	*Lyssavirus formosa*
UK	United Kingdom
WCBV	*Lyssavirus caucasicus*
